# Use of Cooking Fuels and Cataract in a Population-Based Study: The India Eye Disease Study

**DOI:** 10.1289/EHP193

**Published:** 2016-05-25

**Authors:** Thulasiraj D. Ravilla, Sanjeev Gupta, Ravilla D. Ravindran, Praveen Vashist, Tiruvengada Krishnan, Giovanni Maraini, Usha Chakravarthy, Astrid E. Fletcher

**Affiliations:** 1 Lions Aravind Institute of Community Ophthalmology, Aravind Eye Care System, Madurai, India; 2 Centre for Community Medicine, All India Institute of Medical Sciences, New Delhi, India; 3 Aravind Eye Care System, Madurai, India; 4 Dr. Rajendra Prasad Centre for Ophthalmic Sciences, All India Institute of Medical Sciences, New Delhi, India; 5 Aravind Eye Hospital-Pondicherry, Pondicherry, India; 6 Dipartimento di Scienze Otorino-Odonto-Oftalmologiche e Cervico Facciali, Universita` degli Studi di Parma, Parma, Italy; 7 Centre for Vision and Vascular Science, School of Medicine, Dentistry and Biomedical Sciences, Queen’s University Belfast, Belfast, United Kingdom; 8 Faculty of Epidemiology and Population Health, London School of Hygiene & Tropical Medicine, London, United Kingdom

## Abstract

**Background:**

Biomass cooking fuels are commonly used in Indian households, especially by the poorest socioeconomic groups. Cataract is highly prevalent in India and the major cause of vision loss. The evidence on biomass fuels and cataract is limited.

**Objectives:**

To examine the association of biomass cooking fuels with cataract and type of cataract.

**Methods:**

We conducted a population-based study in north and south India using randomly sampled clusters to identify people ≥ 60 years old. Participants were interviewed and asked about cooking fuel use, socioeconomic and lifestyle factors and attended hospital for digital lens imaging (graded using the Lens Opacity Classification System III), anthropometry, and blood collection. Years of use of biomass fuels were estimated and transformed to a standardized normal distribution.

**Results:**

Of the 7,518 people sampled, 94% were interviewed and 83% of these attended the hospital. Sex modified the association between years of biomass fuel use and cataract; the adjusted odds ratio (OR) for a 1-SD increase in years of biomass fuel use and nuclear cataract was 1.04 (95% CI: 0.88, 1.23) for men and 1.28 (95% CI: 1.10, 1.48) for women, p interaction = 0.07. Kerosene use was low (10%). Among women, kerosene use was associated with nuclear (OR = 1.76, 95% CI: 1.04, 2.97) and posterior subcapsular cataract (OR = 1.71, 95% CI: 1.10, 2.64). There was no association among men.

**Conclusions:**

Our results provide robust evidence for the association of biomass fuels with cataract for women but not for men. Our finding for kerosene and cataract among women is novel and requires confirmation in other studies.

**Citation:**

Ravilla TD, Gupta S, Ravindran RD, Vashist P, Krishnan T, Maraini G, Chakravarthy U, Fletcher AE. 2016. Use of cooking fuels and cataract in a population-based study: the India Eye Disease Study. Environ Health Perspect 124:1857–1862; http://dx.doi.org/10.1289/EHP193

## Introduction

Household air pollution (HAP) from combustion of solid fuels for cooking, mainly coal and biomass fuels (wood, crop residues, dung) has been ranked as the second global cause of disability-adjusted life years (DALYs) and a major cause of reduced life expectancy in South Asia in the 2010 Global Burden of Disease (GBD) study (Lim et al. 2012). Causes of morbidity attributed to HAP in the GBD study included cataract although methodological weaknesses in the cited studies (Badrinath et al. 1996; Mohan et al. 1989; Pokhrel et al. 2005; Saha et al. 2005; Sreenivas et al. 1999; Ughade et al. 1998; Zodpey and Ughade 1999) contributing to the GBD estimates were acknowledged. In a later systematic review, including two additional studies (Pokhrel et al. 2013; Tanchangya and Geater 2011), West et al. (2013) concluded that the overall evidence was limited due to selection bias, lack of information on cataract subtypes, categorization of fuel use, and inadequate control for confounding, especially important since biomass fuel use and cataract are associated with a range of poverty-associated factors. Given the widespread use of biomass fuels in India (83% of rural households and 19% of urban) (National Sample Survey Office 2012) and the high prevalence of cataract (Dandona and Dandona 2001; Vashist et al. 2011), further evidence is required on this important topic. We report results from the India age-related eye disease study, a population-based study in two locations in north and south India. Our objectives were to examine risk factors for cataract including biomass fuels.

## Methods

The study sampling has been described in detail previously (Vashist et al. 2011). Briefly, 7,518 people ≥ 60 years old were identified from household enumeration of randomly sampled clusters in north and south India in the catchment area of the following hospitals: Dr. Rajendra Prasad Centre for Ophthalmology in north India (excluding Delhi and Gurgaon) and Aravind Eye Hospital Pondicherry in south India (excluding Pondicherry). All participants gave full informed consent either by signing or a thumb impression. The study complied with the Declaration of Helsinki and ethics approval was received from the All India Institute of Medical Sciences, Aravind Eye Hospital, London School of Hygiene and Tropical Medicine, and Queen’s University Belfast. Data collection took place between September 2004 and December 2006. Fieldworkers interviewed participants at home with a structured questionnaire including demographic and socioeconomic data; tobacco (smoking, chewing) and alcohol use; current and past outdoor work at different times of the day; type of cooking fuels and stoves at three life periods (age of marriage, age of marriage of first child, current); and length of time using fuels and stoves. These descriptors were used to facilitate the recall of the types of fuel and stoves used in early, mid, and late adulthood. Fieldworkers asked respondents about past and present cooking (whether the respondent was the usual cook, age when respondent started cooking, hours respondent spent cooking or hours in the cooking area) and were shown the place where cooking was usually performed. To identify other possible sources of indoor air pollution, the participants were also asked about the type of mosquito repellants and incense they used (Lin et al. 2008; Liu et al. 2003). Within a week of the home interview, participants received a hospital-based clinical examination that included height, weight, mid-upper arm circumference (MUAC), blood pressure, full eye examination, and blood sample collection. Non-fasting capillary blood glucose (CBG) was measured using a reagent strip test and reflectance meter. Vitamin C was measured by automated fluorimetric assay (Vuilleumier and Keck 1989).

### Assessment of Cataract

Following pupillary dilatation, digital nuclear lens images were taken using the Topcon SL-D7 digital slit lamp following a standardized protocol. Retroillumination images of the lens were taken with a Neitz CT-S digital camera to capture cortical and posterior-subcapsular opacities (PSC). Lens opacities were graded by the Lens Opacities Classification System III (LOCS III) (Chylack et al. 1993) in 0.1 unit steps up to 6.9 for nuclear opacities and 5.9 for cortical and PSC. The training and quality assurance of the photographers and graders has been described elsewhere (Vashist et al. 2011).

### Data Preparation and Statistical Analysis

We categorized the type of unoperated cataract based on the LOCS III grade in the worse eye of ≥ 4 for nuclear cataract, ≥ 3 for cortical cataract, and ≥ 2 for PSC. Our definition of “any cataract” included any type of unoperated cataract or with ungradable dense opacities or operated (pseudophakia or aphakia). The comparison group included those with no cataract (i.e., opacity score of < 4 nuclear, and < 3 cortical, and < 2 PSC, and no dense opacities, and no aphakia or pseudophakia). We chose these cut points based on the distribution of LOCS III scores in our participants and to have high sensitivity for visually significant cataract. Household cooking fuels were classified as clean [electricity, liquefied petroleum gas (LPG)] or biomass (wood, crop residues, dung). Kerosene was investigated separately because of concerns of adverse health effects (Lam et al. 2012). Biomass fuel years were calculated by summing years exposed to biomass or clean fuels at each life period. Years of clean fuels were counted as zero years of biomass fuel. Biomass fuel years were transformed to a standardized normal distribution [mean of 0 and standard deviation (SD) of 1]. Principal component analysis was used to derive a socioeconomic status (SES) index (based on caste, landholding, roof type, number of rooms). Categorization of villages and small towns as rural or urban was based on India Census definitions (Census of India 2015). Midday sun exposure was summed over job and life periods and categorized by quartiles. Alcohol and tobacco use were categorized as never or ever. Moderate to severe malnutrition was categorized by MUAC (< 22 in men and < 20 in women) (Ismail and Manandhar 1999). Diabetes was defined as CBG ≥ 110 mg/dL (Somannavar et al. 2009). Plasma vitamin C status was categorized as deficient (< 11 μmol/L) [Institute of Medicine (U.S.) Panel on Dietary Antioxidants and Related Compounds 2000].

Statistical analysis was carried out using Stata (version 13; StataCorp LP, College Station, TX). We investigated potential confounders of biomass fuels and cataract: age, sex, study center, SES, nutritional status [MUAC, body mass index (BMI), vitamin C deficiency], sun exposure, tobacco use, and diabetes. We used multivariable logistic regression to investigate the association of biomass fuel years with cataract or type of cataract, and for comparison with other studies, current use of biomass fuels. We investigated *a priori* hypothesised interactions between biomass fuels with age, sex, center, and vitamin C deficiency. In all analyses, we took account of cluster sampling using linearized variance estimators or robust standard errors.

## Results

Of the 7,518 people ≥ 60 years old identified from enumeration, 7,091 (94%) responded to the questionnaire and 5,871 (83%) of the 7,091 participants underwent an eye exam at the participating hospital. Compared to nonparticipants, the study participants were younger [mean age 68 years (SD = 6)] than the nonparticipants [mean age 70 years (SD = 8)] and less likely to be women (52% and 55%, respectively), but no differences were found in cooking fuel use at any period. Any cataract was defined in 4,098 people (72.7%) of whom 811 had bilateral aphakia or pseudophakia. The unoperated cataracts were nuclear (*n* = 2,404; 1,489 pure, 915 mixed), cortical (*n* = 512; 195 pure, 317 mixed), and PSC (*n* = 1,084; 235 pure, 849 mixed). Of those with nuclear cataracts, 64% remained visually impaired [visual acuity (VA) < 6/18] after best correction for refractive errors; of these, 30% were blind (corrected VA < 3/60). In contrast, only 16% of the non-cataract group had corrected VA < 6/18; of these, 12% were blind.

The mean (SD) of biomass fuel years was 46.6 (11.0). In univariable analysis, older age, lower SES, higher sun exposure, increased years of biomass fuel, tobacco use, and indicators of poor nutrition were more common in those with cataracts while having an inside kitchen and being overweight or diabetic were less common (Table 1). Alcohol was reported by < 1% of women; among men, alcohol use did not differ by cataract status. Biomass fuels declined from 98% (marriage), 89% (marriage of eldest child), and 65% (current) (Table 2). Equivalent figures for LPG were < 1%, 9%, and 32%, respectively. LPG use was lower in the north than in the south. Kerosene was used mainly in the south and was low at all periods. In both the north and south centers, biomass fuels were burnt almost exclusively in a mud or clay stove without a chimney. Kitchen place and current fuel were correlated. Of biomass fuel users, 58% cooked outside the house, 24% in an inside-partitioned area and 18% in an inside-separate kitchen, whereas 95% of LPG users and 93% of kerosene users cooked in a separate inside kitchen. Similar proportions of men and women (65%) reported current biomass fuel use. In age-adjusted linear regression, biomass fuel years were lower among women, participants with diabetes, and those using a separate kitchen and higher in the north, rural participants, those with moderate and severe malnutrition, vitamin C deficiency, tobacco use, and high sun exposure (Table 3). In multivariable analyses, these differences were reduced, for example, from 2 to 1 year fewer biomass fuel years among women than among men. We retained all covariates except for urban and rural residence and alcohol, which were not associated with cataract; BMI was excluded due to collinearity with MUAC.

**Table 1:** Characteristics of study participants by cataract status.

Characteristic^*b*^	No cataract (*n* = 1,500) Mean ± SD or *n* (%)	Any type of cataract or operated cataract^*a*^ (*n* = 4,069) Mean ± SD or *n* (%)	*p-*Value^*c*^
Study center
North India	674 (44.9)	1,972 (48.5)	0.1
South India	826 (55.1)	2,097 (51.5)	
Men	802 (53.5)	1,850 (45.5)	< 0.0001
Rural residence	1,066 (71.1)	3,037 (74.6)	0.06
Age (men)	64.8 ± 4.5	70.1 ± 6.8	< 0.0001
Age (women)	63.3 ± 4.0	67.9 ± 6.4	< 0.0001
Socioeconomic score (fifths)
1 (Lowest)	235 (15.7)	994 (24.4)	< 0.0001
2	252 (16.8)	737 (18.1)	
3	456 (30.4)	1,128 (27.7)	
4	329 (21.9)	674 (16.6)	
5 (Highest)	228 (15.2)	536 (13.2)	
Sun exposure (fourths)
1 (Lowest)	367 (24.5)	1,002 (24.6)	< 0.01
2	431 (28.7)	958 (23.5)	
3	371 (24.7)	1,048 (25.8)	
4 (Highest)	331 (22.1)	1,061 (26.1)	
Ever used tobacco	586 (73.1)	1,497 (80.9)	< 0.0001
Ever used alcohol (men)^*d*^	433 (54.0)	1,027 (55.5)	0.49
Years of biomass fuel use (men)	44.5 ± 10.9	50.2 ± 11.4	< 0.0001
Years of biomass fuel use (women)	40.5 ± 9.4	46.2 ± 9.9	< 0.0001
Kitchen
Outside house	520 (34.7)	1,571 (38.6)	< 0.02
Semi-enclosed kitchen inside house	253 (16.9)	717 (17.6)	
Separate kitchen inside house	727 (48.4)	1,781 (43.8)	
BMI (kg/m^2^)
Underweight (< 18.5)	343 (22.9)	1,392 (34.4)	< 0.0001
Overweight (≥ 25)	358 (23.0)	581 (13.8)	
Vitamin C deficient (< 11 μmol/L)	779 (51.9)	2,504 (61.5)	< 0.0001
Moderate and severe malnutrition^*e*^	136 (9.1)	692 (17.0)	< 0.0001
Diabetes^*f*^	92 (6.1)	190 (4.7)	0.06
^***a***^LOCS III opacity ≥ 4 nuclear, ≥ 3 for cortical, ≥ 2 for posterior subcapsular or dense opacities or cataract surgery. ^***b***^5,569 participants with complete data on all characteristics. ^***c***^*p*-Value for the difference between no cataract and any cataract, univariable analysis. ^***d***^< 1% of women reported any alcohol use. ^***e***^Mid-upper arm circumference of < 22 in men and < 20 in women. ^***f***^Capillary blood glucose ≥ 110 mg/dL.			

**Table 2:** Types of fuels used at three life-periods by study center.

Life event dates	Marriage 1954–1963^*a*^			Marriage of eldest child 1972–1986^*a*^			Current 2005^*a*^		
	North India (*n* = 2,776) *n* (%)	South India (*n* = 3,060) *n* (%)	Both centers (*n* = 5,836) *n* (%)	North India (*n* = 2,776) *n* (%)	South India (*n* = 3,058) *n* (%)	Both centers (*n* = 5,834) *n* (%)	North India (*n* = 2,776) *n* (%)	South India (*n* = 3,028) *n* (%)	Both centers (*n* = 5,804) *n* (%)
Biomass fuels
Wood/crop residues	1,517 (54.6)	1,187 (38.8)	2,704 (46.3)	1,560 (56.2)	1,240 (40.5)	2,800 (48.0)	1,456 (52.4)	1,212 (40.0)	2,668 (46.0)
Wood/crop residues plus dung	947 (34.1)	1,776 (58.0)	2,723 (46.7)	900 (32.4)	1,251 (40.9)	2,151 (36.9)	639 (23.0)	347 (11.5)	986 (17.0)
Dung cakes only	304 (11.0)	0	304 (5.2)	248 (8.9)	0	248 (4.3)	110 (4.0)	0	110 (1.9)
Clean fuels
LPG	0	9 (0.3)	9 (0.2)	65 (2.3)	445 (14.6)	510 (8.7)	567 (20.4)	1,276 (42.1)	1,843 (31.8)
Electricity	0	6 (0.2)	6 (0.1)		5 (0.2)	5 (0.1)
Other fuel
Kerosene	8 (0.3)	82 (2.7)	90 (1.5)	3 (0.1)	117 (3.8)	120 (2.1)	4 (0.1)	193 (6.4)	197 (3.4)
LPG, liquefied petroleum gas. ^***a***^Interquartile range of dates of each life period.									

**Table 3:** Characteristics associated with biomass fuel years.

Characteristic	Difference in biomass fuel years^*a*^ (95% CI)	Difference in biomass fuel years^*b*^ (95% CI)
North vs. south	7.1 (4.9, 9.2)	2.1 (1.1, 3.1)
Women vs. men	–2.1 (–2.8, –1.3)	–1.0 (–1.9, –0.2)
Rural vs. urban	10.1 (8.4, 11.9)	4.6 (3.4, 5.7)
Lowest fifth of socioeconomic score vs. other fifths	3.2 (1.8, 4.4)	1.4 (0.9, 2.0)
Highest fourth of sun exposure vs. other fourths	5.1(3.8, 6.3)	1.8 (1.3, 2.3)
Ever use vs. never use of tobacco	5.7 (4.8, 6.6)	1.5 (1.0, 2.0)
Semi-enclosed kitchen vs. outside	–1.8 (–2.6, –1.0)	–0.7 (–1.3, –0.09)
Separate inside kitchen vs. outside	–11.7 (–13.2, –10.3)	–7.9 (–9.3, –6.5)
Moderate and severe malnutrition^*c*^ vs. normal and mild	3.8 (2.7, 5.0)	0.9 (0.3, 1.5)
Vitamin C deficient (< 11 μmol/L) vs. not deficient	3.7 (2.5, 4.9)	0.8 (0.3, 1.4)
Diabetes^*d*^ vs. no diabetes	–4.7 (–5.4, –3.1)	–0.5 (–1.4, 0.3)
^***a***^Linear regression adjusted for age. ^***b***^Linear regression adjusted for age and all other variables in the table. ^***c***^Mid-upper arm circumference of < 22 for men and < 20 for women. ^***d***^Capillary blood glucose ≥ 110 mg/dL.		

Current use of biomass fuels was associated with an age and sex adjusted odds ratios (ORs) of any cataract of 1.48 [95% confidence interval (CI): 1.24, 1.78] and nuclear cataract of 1.90 (95% CI: 1.55, 2.33). The associations were attenuated after further confounder adjustment [OR of 1.07 (95% CI: 0.90, 1.28) and of 1.24 (95% CI: 1.01, 1.42), respectively. There was no association with cortical cataract or PSC. Sex was an effect modifier of current use of biomass fuels and of years of biomass fuel use (Table 4). For current biomass fuel use the adjusted ORs for nuclear cataract were 1.05 (95% CI: 0.80, 1.38) for men and 1.46 (1.16, 1.84) for women (interaction *p-*value = 0.03).The adjusted ORs for an SD increase in biomass fuel years for any cataract were 0.98 (95% CI: 0.84, 1.14) for men and 1.18 (95% CI: 1.02, 1.36) for women (interaction *p*-value = 0.07); the ORs for nuclear cataract were 1.04 (95% CI: 0.88, 1.23) and 1.28 (95% CI: 1.10, 1.48), respectively (interaction *p*-value = 0.07) (Table 4). There was no evidence for a sex effect for cortical cataract or PSC. The location of the kitchen was not associated with any type of cataract, and the association of current fuel use or biomass fuel years with cataract did not vary by kitchen (*p* = 0.9). We found no interactions with biomass fuel use and any cataract by age (*p* = 0.6), study center (*p* = 0.5), or vitamin C deficiency (*p* = 0.6).

**Table 4:** ORs and 95% CIs of current fuel and biomass fuel years with cataract and cataract type.

Current fuel biomass vs. clean	Any cataract		Nuclear cataract		Cortical cataract		PSC	
	Men (*n* = 2,652)	Women (*n* = 2,917)	Men (*n* = 1,896)	Women (*n* = 1,994)	Men (*n* = 1,021)	Women (*n* = 991)	Men (*n* = 1,281)	Women (1,298)
Current fuel
OR (95% CI)^*a*^	1.34 (1.07, 1.69)	1.64 (1.33, 2.02)	1.65 (1.27, 2.14)	2.18 (1.72, 2.76)	1.24 (0.86, 1.78)	1.31 (0.95, 1.82)	1.14 (0.86, 1.51)	1.22 (0.94,1.58)
*p*-Effect^*b*^	0.012	10^–5^	< 0.0001	10^–5^	0.26	0.10	0.36	0.13
*p*-Interaction^*c*^	0.13		0.05		0.80		0.75	
OR (95% CI)^*d*^	0.96 (0.76, 1.21)	1.20 (0.97, 1.47)	1.05 (0.80, 1.38)	1.46 (1.16, 1.84)	1.04 (0.70, 1.53)	1.10 (0.80, 1.53)	0.88 (0.64, 1.19)	0.99 (0.77, 1.29)
*p*-Effect^*b*^	0.72	0.09	0.71	0.002	0.86	0.55	0.40	0.98
*p*-Interaction^*c*^	0.10		0.03		0.78		0.54	
Biomass fuel years
OR (95% CI)^*d*^^,^^*e*^	0.98 (0.84, 1.14)	1.18 (1.02, 1.36)	1.04 (0.88, 1.23)	1.28 (1.10, 1.48)	1.05 (0.84, 1.33)	1.04 (0.85, 1.27)	0.95 (0.80, 1.13)	1.17 (0.95, 1.44)
*p*-Effect^*b*^	0.82	0.02	0.64	0.001	0.70	0.72	0.56	0.14
*p*-Interaction^*c*^	0.07		0.07		0.88		0.11	
PSC, posterior-subcapsular opacities. ^***a***^Adjusted for age and study center. ^***b***^Effect of biomass fuel use or biomass fuel years on cataract or type of cataract. ^***c***^Difference between men and women in effect of fuel use or biomass fuel years on cataract or type of cataract. ^***d***^Adjusted for age, study center, socioeconomic status, tobacco use, sun exposure, malnutrition, vitamin C deficiency, and diabetes. ^***e***^OR per 1 standard deviation increase in biomass fuel years.								

Only 0.1% of women had never cooked compared to 91% of men; 44% of women (8% of men) cooked in the past but not currently, and 56% of women (1% of men) had always cooked. Of the men who had never cooked, < 6% had spent time in the kitchen area. The average adult lifetime daily hours of cooking by women was shorter among those who had cooked only with biomass fuels (2.4, SD = 0.9) than among those who had used a combination of biomass and clean fuels (2.7, SD = 0.8) (*p* < 0.001). We found no association of cooking hours with cataract in either group: OR = 1.05 (95% CI: 0.90, 1.23) for biomass fuels and OR = 0.88 (95% CI: 0.71, 1.09) for biomass and clean fuels.

Of the 334 participants who reported kerosene use at any period, 321 were from the south center (144 men and 177 women); therefore, analyses were undertaken only for this study center. There was no association of kerosene and cataract outcomes among men (Table 5). However, among women, kerosene was associated with nuclear cataract (OR = 1.76, 95% CI: 1.04, 2.97) and PSC (OR = 1.71, 95% CI: 1.10, 2.64).

**Table 5:** ORs and 95% CIs of any use of kerosene with cataract in South India.

Kerosene use	Any cataract	Nuclear cataract	Cortical cataract	PSC
Men (*n*)	1,378	922	572	667
OR (95% CI)^*a*^	1.10 (0.71,1. 73)	1.38 (0.79, 2.42)	1.32 (0.72, 2.43)	1.30 (0.74, 2.31)
*p*-Effect^*b*^	0.67	0.26	0.37	0.36
Women (*n*)	1,545	1,016	562	674
OR (95% CI)^*a*^	1.52 (0.98, 2.36)	1.76 (1.04, 2.97)	1.31 (0.82, 2.09)	1.71 (1.10, 2.64)
*p*-Effect^*b*^	0.06	0.04	0.25	0.02
*p*-Interaction^*c*^	0.26	0.52	0.99	0.48
PSC, posterior-subcapsular opacities. ^***a***^Adjusted for age, years of biomass fuel use, socioeconomic status, tobacco use, sun exposure, malnutrition, vitamin C, diabetes. ^***b***^Effect of kerosene use on cataract or type of cataract. ^***c***^Difference between men and women in effect of kerosene use on cataract or type of cataract.				

The use of mosquito repellants differed by study center. Overall, 40% of participants: 55% in the south and 23% in the north, reported any use, but a smaller proportion (30% and 4%, respectively) used them on a regular basis. The median years of repellant use was 5 for regular all-year-round use and 3 years for occasional or seasonal use. For those who used repellants, the most common types in both the south and north study centers were mosquito coils (71% in the south and 36% in the north) and vaporizers (24% and 35%, respectively); mosquito mats (4% and 10%, respectively) were used less frequently. In addition, < 1% of the participants in the south used crop residues or dung as repellants compared to 10% and 9%, respectively, in the north. In both centers, vaporizer use was more common among higher SES and urban participants, whereas the inverse was observed for coils. The average daily use of crop residues was 5 hr and 1 hr for dung. Use of mosquito coils was not associated with any cataract or cataract type, either in comparison with non-users of mosquito repellants, OR = 0.91 (0.78, 1.05), or in comparison with other types of repellants, OR = 1.05 (0.88, 1.24). Daily use of incense was reported by 28% and 57% in the south and north respectively. There was no association with incense and any cataract, OR = 0.86 (0.70, 1.05) or with any type of cataract. Inclusion of mosquito and incense use did not influence the estimates for biomass fuel use.

## Discussion

We found that exposure to biomass fuels over adult lifetime was associated with cataract in women but not in men. The ORs for nuclear cataract were 1.28 (1.10, 1.48) per one SD increase in lifetime years of biomass exposure. There was no association with cortical or PSC. The association with women is plausible since cooking was almost exclusively done by women using stoves without chimneys, thus exposing them directly to smoke from combustion of biomass fuels. We found no association with cataract of daily time spent cooking using biomass fuels. Poor recall of time spent cooking especially in a largely rural and resource-poor setting may account for the lack of any relationship. We found that kitchen place did not influence the association of biomass fuel with cataract but we collected data on kitchen place only at survey.

Studies from Indian regions (Balakrishnan et al. 2011) along with data on PM_2.5_ collected for the GBD study (Balakrishnan et al. 2013) have shown high levels of respirable particulates from biomass fuels, especially the most damaging PM_2.5._ Levels were highest in kitchens, in poorly ventilated areas and during cooking. Average 24-hr PM_2.5_ exposures from regression modeling (based on measurements in kitchen and living areas using different fuels, stoves, cooking duration) were 285 μg/m^3^, 337 μg/m^3^, and 204 μg/m^3^ for children, women and men respectively (Balakrishnan et al. 2013). Men had high PM_2.5_ levels although much lower than those for women. Levels were substantially higher in kitchen compared to living areas, e.g. for wood users, medians of 386 and 87 respectively. We did not collect data on time spent in living areas. Only a small proportion of non-cooking men (6%) reported spending time in the kitchen during cooking suggesting that men in our study were less exposed to biomass fuel smoke.

Studies in India have reported adverse effects of particles on physiological measures (Dutta et al. 2012; Mudway et al. 2005; Padhy and Padhi 2009). Compared to women using LPG, women cooking with biomass fuels were exposed to three times the levels of PM_10_ and PM_2.5_, a 37% increase in reactive oxygen species (ROS) and a 40% depletion of the antioxidant enzyme superoxide dismutase (SOD); PM_10_ and PM_2.5_ levels were positively associated with ROS (Dutta et al. 2012). Children in biomass fuel, compared to LPG, households had substantially lower concentrations of serum ascorbate, lower SOD and a higher ratio of glutathione (GSH) to oxidized GSH, a measure of oxidative stress (Padhy and Padhi 2009). In a synthetic model of human respiratory tract lining fluid incubated with particles from dung combustion, ascorbate was depleted by 70–90% over a 4-hr period and GSH by 50–60% dependent on the particle concentration (Mudway et al. 2005). Iron and copper in the particles were likely to be the main sources of redox activity. Findings from these studies are particularly relevant to our results of the association of biomass fuels with nuclear cataract. GSH and ascorbate are the principal lens antioxidants and protect the nuclear lens proteins from the highly reactive hydroxl radical generated by redox active metals, principally iron (Fu et al. 1998; Garner et al. 2000; Truscott 2005). Plasma ascorbate concentrations and dietary intakes of vitamin C were very low in our study population (Ravindran et al. 2011). The association with nuclear cataract in women may be explained by increased exposure to redox active metals through combustion of biomass fuels and inadequate antioxidant protection by vitamin C. We found no effect modification of biomass fuel use by plasma ascorbate deficiency. However vitamin C was measured at survey and may not reflect lifetime vitamin C levels.

Other constituents of biomass fuel combustion are polycyclic aromatic hydrocarbons (PAH) (including benzene, toluene, naphthalene). Naphthalene is of particular interest due to its use as a cataract inducer in experimental studies with rabbit and rodent lenses (Van Heyningen and Pirie 1967). The main effect of naphthalene in the lens is depletion of glutathione and production of ROS (Stohs et al. 2002). Whether these studies of acute high oral doses of naphthalene can be extrapolated to cataract development in humans is uncertain especially since chronic low level dosing of rats has not led to lens opacities (WHO 2010). An early case-report of lens opacification in young men occupationally exposed to high levels of naphthalene (Ghetti and Mariani 1956) has not been confirmed. There are limited data on indoor levels of naphthalene from biomass fuel combustion in low and middle-income countries and none from India. A study in Burundi of wood for cooking found that naphthalene was the main indoor PAH constituent (around 70%) with very high levels of 29 μg/m (SD = 23) (Viau et al. 2000) compared to typical values of 1–2 μg/m in European studies (WHO 2010). Mosquito coils and incense are also important sources of indoor naphthalene emissions and high levels have been shown in East Asian settings (Lin et al. 2008; Liu et al. 2003). No association of mosquito coils or incense with cataract was found in the two studies that investigated these exposures (Pokhrel et al. 2005, 2013). We also did not find any association with cataract which may reflect lower naphthalene emissions of repellents or incense in our population, poor recall of these exposures or a true lack of effect. A WHO report in 2010 concluded that with respect to cataract there was only suggestive evidence of an association with exposure to naphthalene in humans, if at all (WHO 2010).

Kerosene was not commonly used for cooking in our study (6% overall), comparable to census data (National Sample Survey Office 2012). We did not collect data on kerosene for lighting. We found an association in women with kerosene and nuclear cataract or PSC but no association in men. These differences warrant caution since interaction *p*-values were 0.5. Little information is available on emissions and health outcomes from cooking with kerosene (Lam et al. 2012; WHO 2014). A recent review by WHO recommended that kerosene should not be used as a household fuel due to concerns about emissions and safety (WHO 2014). Studies in India showed concentrations of respirable particulates varied according to the type and location of the kerosene stove, length of cooking time and position of the cook (Balakrishnan et al. 2013; Raiyani et al. 1993; Saksena et al. 2003; Smith et al. 2000) but were substantially lower than those for biomass fuels. Saksena et al. (2003) found the average kitchen concentrations of particulates were lower in kerosene compared to wood users but personal measures were similar and concluded that cooking duration, use of an indoor kitchen and proximity to the stove were explanatory factors. In our study, 94% of current kerosene fuel users used a separate indoor kitchen. Other toxic emissions from inefficient kerosene stoves in India include PAHs, in particular naphthalene (Pandit et al. 2001). No association with cataract was found with kerosene either for lighting (Pokhrel et al. 2005) or for cooking (Pokhrel et al. 2013).

Previous studies of biomass fuels and cataract have been summarized in two meta-analyses based on 7 (Smith et al. 2014) and 12 studies (Kulkarni et al. 2014): 7 of the studies were in both meta-analyses, with summary effect sizes of 2.46 (95% CI: 1.74, 3.30) and 2.12 (95% CI: 1.61, 2.80), respectively, for current use of biomass fuels. Another recent study was conducted in Nepal (Pokhrel et al. 2013). All studies were conducted in a South Asian setting. While most (but not all) studies found an association with biomass fuels and cataract, there were sufficient limitations in the design and analysis to warrant closer scrutiny. The majority were hospital-based studies (Badrinath et al. 1996; Mohan et al. 1989; Pokhrel et al. 2005, 2013; Saha et al. 2005; Tanchangya and Geater 2011; Ughade et al. 1998; Zodpey and Ughade 1999) with hospital-based controls attending ophthalmology clinics. Lack of information on the selection of cases and, in particular controls, including control diagnoses and response rates, raises concerns of selection bias especially in hospital based-studies. Cataract was determined by clinical assessment in most studies with little information on criteria or severity. Only 3 studies used a lens opacity classification system (Krishnaiah et al. 2005; Mohan et al. 1989; Pokhrel et al. 2013). Some studies included only women (Pokhrel et al. 2005, 2013; Zodpey and Ughade 1999) or asked about cooking fuels in women only (Krishnaiah et al. 2005). One study alone provided separate estimates for men and women (Tanchangya and Geater 2011), but these were based on different comparisons of cooking fuel. Most studies collected data on current cooking fuels only and 2 studies also investigated previous use (Pokhrel et al. 2013; Tanchangya and Geater 2011). Studies compared biomass or solid fuels with LPG (Mohan et al. 1989; Pokhrel et al. 2013; Saha et al. 2005) or with LPG plus kerosene (Krishnaiah et al. 2005; Pokhrel et al. 2005; Sreenivas et al. 1999). Most studies were small and may not have adequately adjusted for confounding especially those that did not match for age. We found no association of biomass fuels with cortical or PSC cataract. Of the 2 studies that investigated cataract type (Mohan et al. 1989; Pokhrel et al. 2013), only 1 found an association with pure cortical and with mixed cataracts (Mohan et al. 1989).

The strengths of our study were a large random population-based sample, ascertainment of cataract by digital lens images with a well-established classification system, and detailed information on key confounders. Participants underwent an eye examination thus minimizing selection bias since cataract was measured in the same population and without knowledge of biomass fuel exposure. We cannot exclude bias due to nonresponse to the eye exam. Participants were slightly younger and male compared to nonparticipants. We aimed to measure long-term biomass fuel use to provide a more valid estimate of exposure than current fuel which reflects recent adoption of clean fuels in India. Past fuel use, especially in mid-adulthood, and duration of different fuel use may be inaccurately reported. These recall errors are probably nondifferential (i.e., not biased by knowledge of cataract status) and likely to attenuate the association of fuel use and cataract. Recall errors might be greater in men than women (since men rarely cook) and could account for the lack of an association in men. Our information on kitchens was limited to current kitchens without details of ventilation or proximity to living and sleeping areas. We adjusted for other sources of indoor air pollution (mosquito repellants and incense), but did not collect data on passive smoking.

Our results for long-term exposure to biomass fuels apply to a binary measure of visually significant cataract. We chose this outcome as the most relevant to the GBD study. Previous studies in India have shown that inability to work or do household tasks, reduced social participation, feelings of isolation, and being a burden are consequences of cataract (Fletcher et al. 1998, 1999). In future analyses, we plan to explore the relationship between biomass fuel years and lens opacity score as a guide to how progression of mild lens opacities might be reduced by change in fuel use. We will further model our results in terms of PM_2.5_ exposures using published estimates for India (e.g., by fuel and kitchen type) (Balakrishnan et al. 2013).

## Conclusions

Our results provide robust evidence for the association of biomass fuels with nuclear cataract in women but not in men. Our finding of an association with kerosene use in women is novel but requires confirmation in other studies. Our study, like all previous studies, took place in South Asia limiting the generalizability of our results to other settings with high use of biomass fuels but different cultural and lifestyle factors including the involvement of men in cooking.
